# Supplementary material for: Genetic Diversity Assessment and Core Germplasm Screening of Blackcurrant (Ribes nigrum) in China via Expressed Sequence Tag–Simple Sequence Repeat Markers
Source: Int J Mol Sci. 2025 Mar 6;26(5):2346. doi: 10.3390/ijms26052346 (PMC11899734; doi:10.3390/ijms26052346)
Supplement: Supplementary file 1 [file ijms-26-02346-s001.zip › Figure S1.pdf]

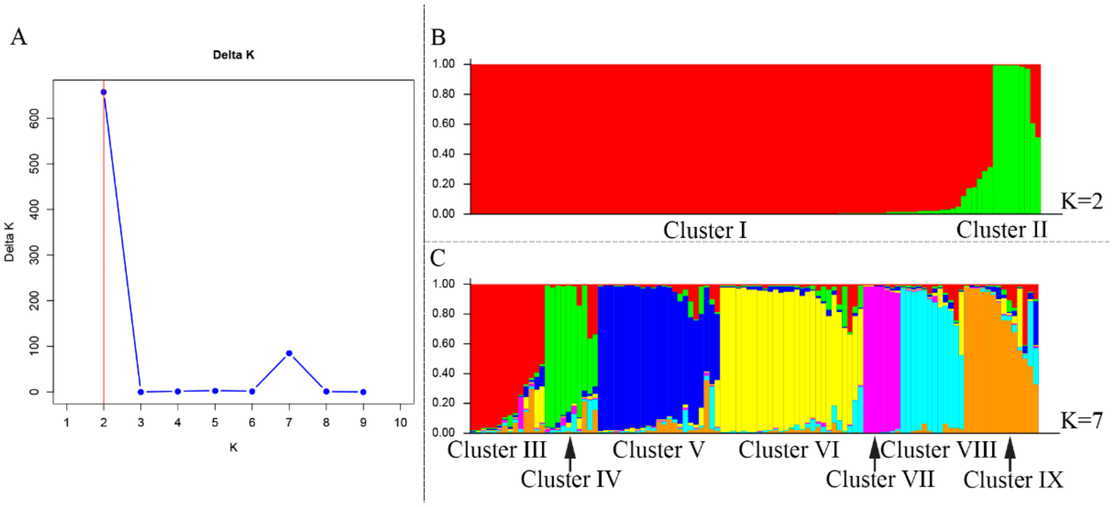

Figure S1. Population structure analysis of 107 *Ribes* accessions based on 31 EST-SSR markers. A, Delta K calculation was performed for each K value according to the Evanno method; B, Population structure analysis (K = 2); C, Population structure analysis (K = 7).
