# Supplementary material for: Genetic Diversity Assessment and Core Germplasm Screening of Blackcurrant (Ribes nigrum) in China via Expressed Sequence Tag–Simple Sequence Repeat Markers
Source: Int J Mol Sci. 2025 Mar 6;26(5):2346. doi: 10.3390/ijms26052346 (PMC11899734; doi:10.3390/ijms26052346)
Supplement: Supplementary file 1 [file ijms-26-02346-s001.zip › Table S3-S7.pdf]

Table S3. Population strucrture analysis classification of 95 blackcurrant cultivars at K=3.

Cluster	Accessions name
Cluster A	Ojebyn, Orville, Zusha, Exotic, Globus, Gejinzige, Adelinia, Big Ben, Sophia, Lama, Ben Gairn, Bagada, Bagira, Bona, Kantata, Suiyanyihao, E-15, E16, 16A, 14(17-5), A16, Nailor, Hanfeng, 14C, 16C, 18C, Aw-2, 15-3, 17-29, 17-29-3, Muxuan 2008-6, Muxuan 2013-10, Muxuan 2015-13
Cluster B	Risager, Roodknop, Ben Nevis, Baldwin, Mendip Cross, Black smith, Fertodi, Liangyehoupi, Xielieqinaya, Belaruskaja, Ben Tirran, Zwiezda, Gezishiseng, Primorskij pearl, 0A14, Suiyanerhao, Danjianghei, Suanpanzi, Baopifengchan, C17, C11, C19, C28, 94-4-13, W1-2, 17B, 13C, 15C, 17C, E-14, Muxuan 2012-6
Cluster C	Vologda, Ben Lomond, 19C, Aw-4, Aw-3, Aw-1, BW-2, BW-3, Bw-4, Yade-1, Yade-2, Yade-3, Yade-4, 15-1, 15-2, 15-4, 15-5, 15-6, 15-7, 15-8, 15-9, 15-10, Bw-1, Lw-1, 17-29-1, 17-29-2, 17-29-4, 17-29-5, SU-3, Muxuan 2011-14, Muxuan 2015-10

Table S4. Population strucrture analysis classification of 95 blackcurrant cultivars at K=5.

Cluster	Accessions name
Cluster D	Liangyehoupi, Xielieqinaya, Vologda, Globus, Zwiezda, Gezishiseng, Bona, Danjianghei, C11, 94-4-13, 17-29, A16, 18C, 17B
Cluster E	Ojebyn, Gejinzige, Adelinia, Big Ben, Sophia, Lama, Ben Gairn, Kantata, Suiyanyihao, E16, 16A, 13C, 14C, 15C, 19C, Aw-2, Bw-4
Cluster F	Zusha, Exotic, Bagada, Belaruskaja, Nailor, C19, W1-2, 17C, 0A14, E-15, 15-6, 14(17-5), Muxuan 2008-6, Muxuan 2013-10, Muxuan 2015-13
Cluster G	Risager, Roodknop, Ben Nevis, Baldwin, Mendip Cross, Black smith, Fertodi, Orville, Ben Tirran, Bagira, Ben Lomond, Primorskij pearl, Suiyanerhao, Suanpanzi, Baopifengchan, C17, C28, 16C, BW-3, E-14, Muxuan 2012-6, Muxuan 2015-10
Cluster H	Hanfeng, BW-2, Aw-1, Aw-3, Aw-4, Bw-1, Lw-1, Yade-1, Yade-2, Yade-3, Yade-4, 15-1, 15-2, 15-3, 15-4, 15-5, 15-7, 15-8, 15-9, 15-10, 17-29-1, 17-29-2, 17-29-3, 17-29-4, 17-29-5, SU-3, Muxuan 2011-14

Table S5. Population strucrture analysis classification of 107 *Ribes* accessions at K=2.

Group	Species	Accessions number	Accessions name
Cluster I	*Ribes nigrum*	95	Adelinia, Bagada, Bagira, Baldwin, Baopifengchan, Belaruskaja, Ben Gairn, Ben Lomond, Ben Nevis, Ben Tirran, Big Ben, Black smith, Bona, Exotic, Danjianghei, Fertodi, Gejinzige, Gezishiseng, Globus, Hanfeng, Kantata, Lama, Liangyehoupi, Mendip Cross, Nailor, Ojebyn, Orville, Primorskij pearl, Risager, Roodknop, Suanpanzi, Suiyanyihao, Suiyanerhao, Xielieqinaya, Vologda, Sophia, Zusha, Zwiezda, C17, C19, C28, C11, 94-4-13, 17-29, W1-2, E16, 16A, 14(17-5), A16, 19C, 18C, 17B, 13C, 14C, 15C, 16C, 17C, 15-3, BW-2, 15-1, 15-2, 15-4, 15-5, 15-6, 15-7, 15-8, 15-9, 17-29-1, 17-29-2, 17-29-3, 17-29-4, 17-29-5, Aw-1, Aw-2, Aw-3, Aw-4, 15-10, Bw-1, BW-3, Bw-4, Yade-1, Yade-2, Yade-3, Yade-4, Lw-1, 0A14, SU-3, E-14, E-15, Muxuan 2008-6, Muxuan 2012-6, Muxuan 2015-10, Muxuan 2011-14, Muxuan 2013-10, Muxuan 2015-13
Cluster II	*Ribes ussuriensis*	1	Ussuri
	Ribes uva-crispa	1	Pixwell
	Ribes panciflorum	1	Xinganchabiao
	Ribes rubrum	7	Witte Parel, Crusader, Cherry, ER-1, Maer, Red Spring, E-RED
	Ribes americanum	1	Hongyeheidou
	Ribes odoratum	1	Xiangchabiaozi

Table S6. Population strucrture analysis classification of 107 *Ribes* accessions at K=7.

Group	Species	Accessions number	Accessions name
Cluster III	*Ribes nigrum*	14	Ojebyn, Gejinzige, Big Ben, Lama, Bona, E16, 16A, 19C, 13C, 14C, 15C, Aw-2, Yade-3, SU-3
Cluster IV	*Ribes nigrum*	7	Globus, Bagada, 17-29, 14(17-5), 17C, 0A14, Baopifengchan
	Ribes americanum	1	Hongyeheidou
	Ribes uva-crispa	1	Pixwell
	Ribes odoratum	1	Xiangchabiaozi
Cluster V	*Ribes nigrum*	22	Risager, Roodknop, Ben Nevis, Baldwin, Mendip Cross, Black smith, Fertodi, C17, Liangyehoupi, Belaruskaja, Ben Tirran, C19, W1-2, 16C, Ben Lomond, Primorskij pearl, Suiyanyihao, Danjianghei, Suanpanzi, E-15, Muxuan 2008-6, Muxuan 2012-6
	Ribes panciflorum	1	Xinganchabiao
Cluster VI	*Ribes nigrum*	27	Hanfeng, 15-3, 17-29-1, 15-4, BW-2, 15-2, 15-1, Aw-4, Aw-3, 17-29-3, 15-8, Aw-1, 15-10, 17-29-2, 17-29-5, 15-6, BW-3, Yade-1, Yade-2, 15-5, Bw-1, Lw-1, 15-9, 17-29-4, Yade-4, Bw-4, 15-7
Cluster VII	*Ribes rubrum*	7	Witte Parel, Crusader, Cherry, ER-1, Maer, Red Spring, E-RED
Cluster VIII	*Ribes nigrum*	11	Orville, Zusha, Adelinia, Sophia, Ben Gairn, Kantata, E-14, Muxuan 2015-10, Muxuan 2011-14, Muxuan 2013-10, Muxuan 2015-13
	Ribes ussuriensis	1	Ussuri
Cluster IX	*Ribes nigrum*	14	Exotic, Xielieqinaya, Vologda, Bagira, Zwiezda, Gezishiseng, C28, C11, 94-4-13, A16, Nailor, 18C, 17B, Suiyanerhao

Table S7. Comparison of blackcurrant cultivars of core sets constructed by different methods.

Type	Accessions name
PC	Vologda, Bona, Ben Lomond, Primorskij pearl, Kantata, Suiyanyihao, Suiyanerhao, Danjianghei, Hanfeng, Bw-4, Yade-1, Yade-4, C11, 17-29, W1-2, E16, 14C, 15-1, 15-10, Muxuan 2008-6, Muxuan 2015-13
CH	Vologda, Ben Lomond, Primorskij pearl, Ojebyn, Ben Gairn, Bagada, Suiyanyihao, Danjianghei, Hanfeng, Aw-4, Bw-4, Yade-1, Yade-4, E-15, E16, 14C, 15-10, Muxuan 2008-6, Muxuan 2015-10
IC	Vologda, Bona, Ben Lomond, Primorskij pearl, Ojebyn, Ben Gairn, Bagada, Kantata, Hanfeng, Suiyanyihao, Suiyanerhao, Danjianghei, Aw-4, Bw-4, Yade-1, Yade-4, E-15, E16, C11, 17-29, W1-2, 14C, 15-1, 15-10, Muxuan 2008-6, Muxuan 2015-10, Muxuan 2015-13
